# Asymptomatic Thoracic Migration of a Ventriculoperitoneal Shunt: A Case Report

**DOI:** 10.7759/cureus.69683

**Published:** 2024-09-18

**Authors:** Jackson Griffith-Linsley, Matthew P Blackwell, Dustin J Gulizia

**Affiliations:** 1 Medical School, Indiana University School of Medicine, Indianapolis, USA; 2 Interventional Radiology, Rush University Medical Center, Chicago, USA

**Keywords:** distal catheter migration, shunt complications, ventriculoperitoneal shunt, vps, vp shunt

## Abstract

Hydrocephalus is often treated with CSF diversion via ventriculoperitoneal (VP) shunting. We present the unique case of a 33-year-old female with a history of infiltrating astrocytoma and consequent obstructive hydrocephalus necessitating shunt placement. She later presented with non-specific symptoms prompting shunt evaluation. Ultimately, while a cause for her symptoms was not identified, imaging revealed distal catheter migration into the pleural space. The patient remained asymptomatic during two years of follow-up without surgical intervention. This case highlights the potential for asymptomatic distal catheter migration after VP shunt placement and underscores the importance of appropriate monitoring and management once such migration is detected.

## Introduction

Hydrocephalus refers to the over-accumulation of cerebrospinal fluid (CSF) within the cerebral ventricular system [1]. Though etiologies are varied, clinical presentation generally includes some combination of confusion, disorientation, drowsiness, lethargy, irritability, headache, nausea, vomiting, decreased appetite, and vision changes [1]. Treatment involves shunting the CSF to another location, most commonly via a ventriculoperitoneal (VP) shunt [1]. In a VP shunt, the proximal catheter drains excess CSF from the ventricles of the brain, while the distal catheter is tunneled subcutaneously through the anterior or anterolateral chest wall and into the subcutaneous tissues of the abdomen. It typically enters the peritoneal cavity in the right or left lower quadrant of the abdomen, where the CSF is absorbed by the body. Although generally considered safe and effective, complications related to VP shunts are not infrequent [1]. A recent review reported that 23.8% of patients experienced at least one shunt-related complication during a mean follow-up time of only 3.9 years [2]. These include infection, hemorrhage, obstruction, fracture/disconnection, and migration, in addition to complications specific to the site of distal catheter placement (e.g. intra-peritoneal, pleural, atrial, etc.) [2]. Thoracic migration of a VP shunt is a rare complication that is sparsely covered in the literature but can be the source of significant concern on initial diagnosis. Here we present the case of an adult female with a VP shunt complicated by distal catheter migration into the right hemithorax.

## Case presentation

A 33-year-old female with a history of grade II infiltrating astrocytoma of the bilateral thalami complicated by obstructive hydrocephalus and post-VP shunt placement one year prior presented to the emergency department (ED) with a seven-day history of headache, nausea, vomiting, somnolence, and memory and cognition changes. Examination revealed intermittent alertness with increased speech latency and single-word responses to questions. The initial workup, which included vital signs and laboratory results, was unremarkable. Due to the known tumor burden, a dose of intravenous dexamethasone was given, with minimal symptomatic improvement. She was admitted to neurology for further evaluation and treatment.

**Figure 1:**
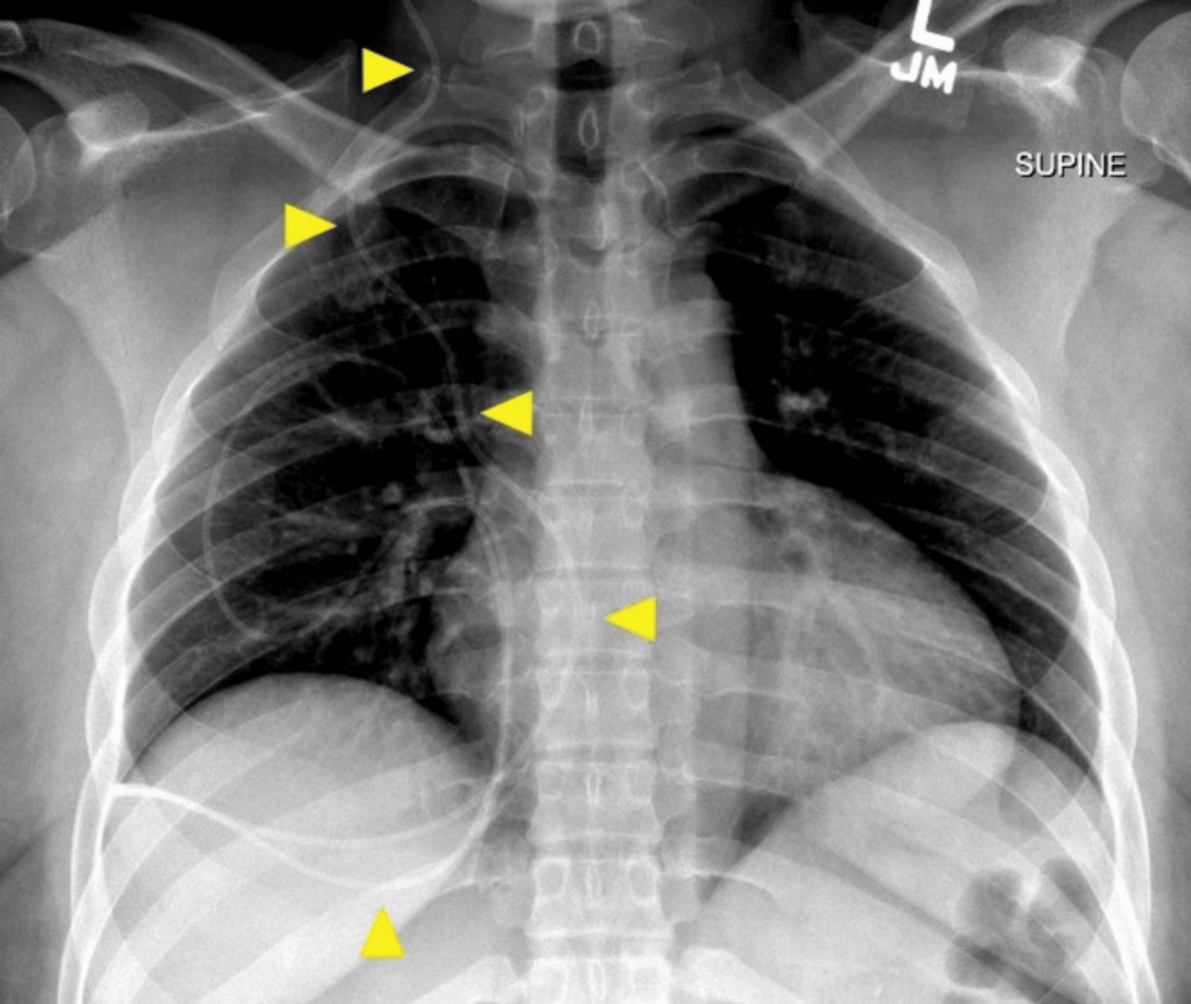
Ventriculoperitoneal (VP) Shunt Series on Emergency Department Presentation Frontal chest radiograph obtained as part of VP shunt series during workup in ED demonstrates looping and curling of the distal VP shunt catheter (yellow arrowhead) over the right hemithorax.

**Figure 2:**
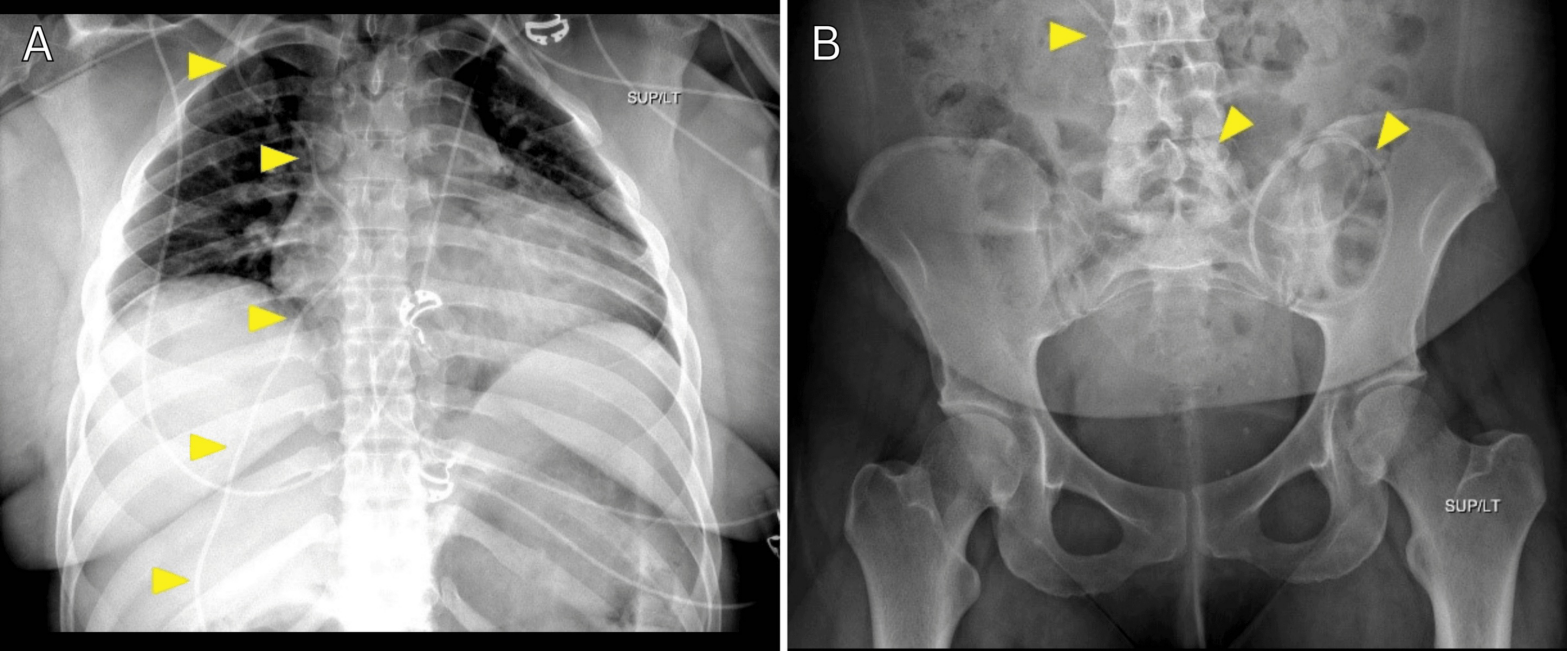
Ventriculoperitoneal (VP) Shunt Series After Initial Placement Frontal chest (A) and abdominal (B) radiographs obtained as part of the VP shunt series immediately after placement demonstrate a normal course of the VP shunt catheter (yellow arrowhead) over the right hemithorax, with distal tip curling over the left lower quadrant of the abdomen.

**Figure 3:**
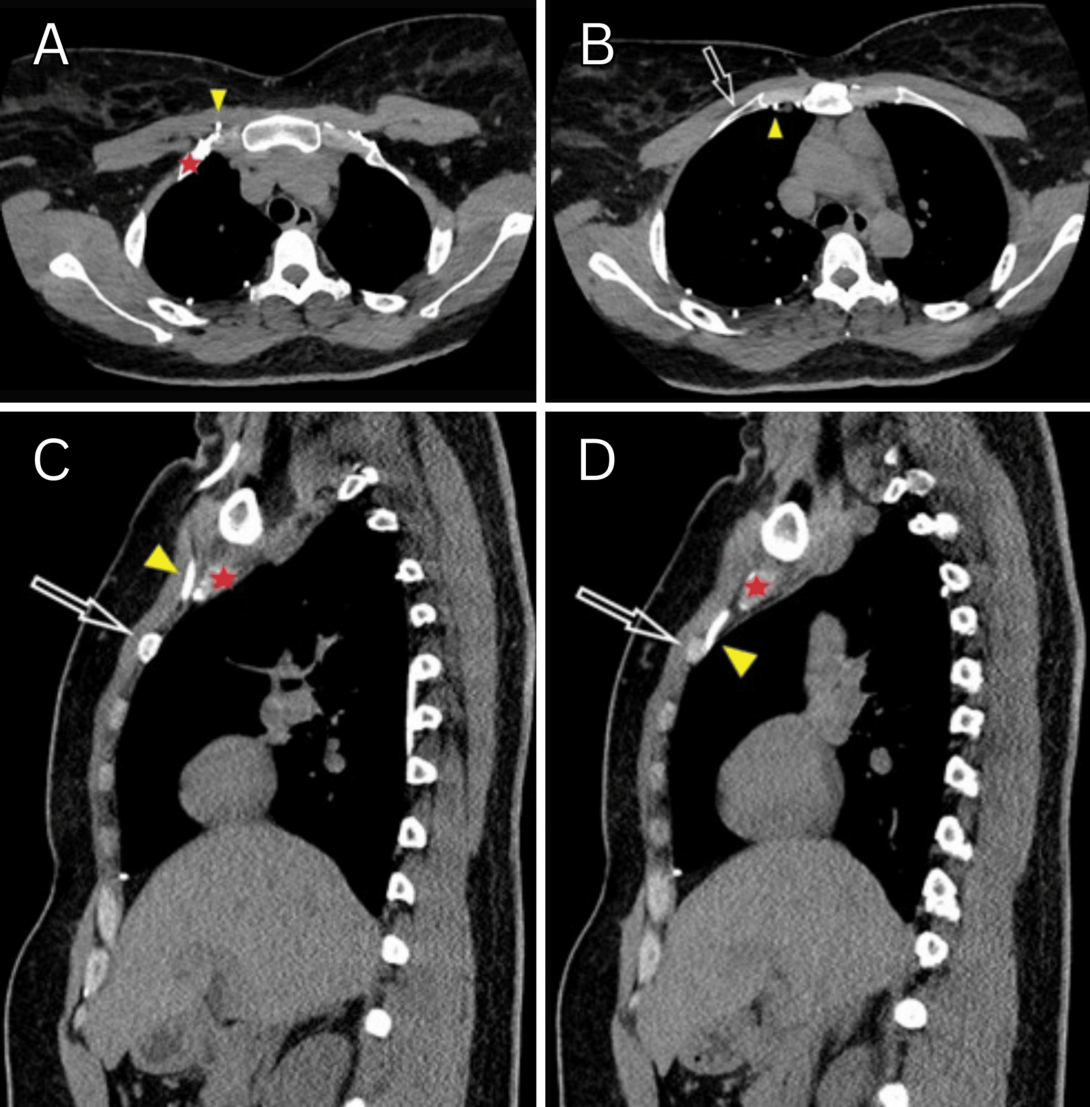
Chest Computed Tomography Sequential CT images of the axial series (A-B) and sagittal series (C-D) demonstrate the VP shunt catheter (yellow arrowhead) coursing superficially over the right first rib (red star) before entering the pleural space and coursing deep to the right second rib (open white arrow).

Magnetic resonance imaging (MRI) of the brain obtained the following day was stable compared to the previous with no significant increase in tumor burden, cerebral edema, or hydrocephalus. The radiographic shunt series, however, revealed interval migration of the distal catheter into the right hemithorax (Figure 1) when compared to postoperative radiographs (Figures 2A, 2B). Follow-up computed tomography (CT) chest confirmed the location of the distal catheter within the pleural space (Figures 3A-3D), a clear departure from imaging obtained one year prior, which demonstrated appropriate location within the abdomen. Interrogation of all shunt components, including the valve, shunt reservoir, and proximal/distal catheters by neurosurgery, confirmed the shunt was intact and functional. Ultimately, no cause was identified for the patient’s presenting symptoms, and she was subsequently discharged with no significant changes to management after symptom resolution.

Shunt location and function were monitored via serial imaging over the next two years without any changes. Despite the intrapleural location of the distal catheter, the patient continued to have no clinically significant symptoms as a result of migration.

## Discussion

Ventricular shunting is a common procedure to alleviate significantly morbid symptoms of hydrocephalus. Numerous types of shunts exist, including the VP shunt, ventriculopleural shunt, and ventriculoatrial shunt [3].

Although each shunt has benefits and drawbacks, the VP shunt comprises the majority of shunts in place today. The peritoneal epithelium has a high potential for fluid absorption and thus makes an ideal place for CSF shunting [4]. In a similar fashion, the pleura may also absorb fluids. However, when over-capacitated, clinically significant hydrothorax may develop and, in more serious cases, result in cardiopulmonary compromise [5-8]. Ventriculoatrial or other vascular shunts are also pursued at times, but are not preferred due to the potential for morbid bloodstream infection and thrombosis, among other severe complications [9].

VP shunts are also known to have complications, with the majority occurring within the first year of placement and up to 80% of patients requiring at least one shunt revision over the course of their lifetime [2,10,11]. Reported complications beyond infection, obstruction, and breakage include pseudocyst formation, intestinal obstruction, incisional hernia, and catheter migration [12].

Catheter migration of the distal tip, though rare, is a well-described complication. Migration into the pleural space may be supradiaphragmatic or transdiaphragmatic [13]. Transdiaphragmatic migrations are thought to arise due to the natural variant diaphragmatic foramen of Bochdalek or Morgangi, or inflammatory erosion of the diaphragm [14]. Supradiaphragmatic catheter migrations are most likely secondary to procedural technique with unintentional passage into the pleural space while tunneling through the subcutaneous tissues during placement. Over time, the negative pressure within the thoracic cavity during inspiration may result in the retraction of the catheter into the pleural cavity. Likewise, if there is an unintentional passage through a vein during tunneling, with prolonged intermittent negative pressure, the catheter may migrate into the central veins, right heart chambers, or pulmonary arteries [15,16]. Other causes of distal catheter migration have also been suggested. Tamura et al. discussed the potential for repetitive flexion and extension of the neck to enable proximal movement of the peritoneal catheter via the “windlass effect” [17-20]. Pang and Wilberger and Kim et al. reported cases of potential space formation during the subgaleal and subcutaneous tunneling process, allowing for coiling and distal catheter migration [17-19]. Appropriate anchoring of the shunt system and careful dissection through connective tissues can help minimize these risk factors.

In our case, the catheter is noted to enter the pleural space between the first and second rib on CT imaging (Figure 3). Most commonly, this would be suspected after a demonstration of pneumothorax on immediate postoperative shunt series radiographs. However, if there is no associated lung injury, a pneumothorax may not develop and the suboptimal catheter trajectory may not be known for months to years until revealed by repeat imaging or the advent of complications.

The migration into the pleural space was asymptomatic in this case, and not associated with the patient's clinical presentation. However, if the pleural lining is no longer able to maintain adequate absorption, symptomatic hydrothorax may arise in a delayed fashion [3,6]. Alternatively, other complications such as pulmonary edema, bronchial perforation, pneumonia, and respiratory distress may arise [14,15]. Knowledge of these potential complications is important for any physician who may encounter VP shunts in practice, including emergency physicians, neurosurgeons, neurologists, pediatricians, and radiologists. This will allow for appropriate monitoring of the patient and prevention of worsening morbidity or even mortality.

## Conclusions

This case report highlights the importance of recognizing and managing a rare complication of VP shunts: distal catheter migration into the pleural space. The patient's clinical course, characterized by nonspecific symptoms and an asymptomatic intrapleural catheter, serves as a valuable lesson for healthcare providers involved in the care of patients with VP shunts. Prompt and thorough evaluation, including appropriate imaging, is essential to identify complications even when initial clinical assessments are inconclusive.

Additionally, while this case does not suggest the need for routine follow-up imaging in all patients with VP shunts, it demonstrates the importance of adjusting follow-up when complications, such as a pleural defect, are identified on post-operative imaging. If a pleural defect is detected, a follow-up shunt series, including lateral chest views, at three to six months may be warranted to monitor for potential migration and ensure catheter stability, with additional follow-up as indicated. If migration occurs later, targeted imaging may be necessary to monitor for pleural effusion, assess catheter stability, and detect complications such as hydrocephalus or slit ventricles, even in asymptomatic patients.
